# Selective single blastocyst transfer study: 604 cases in 6 years

**DOI:** 10.4103/0974-1208.39591

**Published:** 2008

**Authors:** Nirmala Sadasivam, Narayanan M. Sadasivam

**Affiliations:** Department of Infertility Medicine, Maaruthi Medical centre and Hospitals, Erode, Tamilnadu, India

**Keywords:** Ovarian hyper stimulation syndrome (OHSS), single blastocyst transfer (SBT), single embryo transfer (SET)

## Abstract

**AIM:**

To evaluate the credibility of single blastocyst transfer (SBT) method in selected group of patients.

**SETTINGS AND DESIGN:**

Retrospective analysis of SBT cases based on computerized data in a private Fertility research centre.

**MATERIALS AND METHODS:**

A total of 604 cases of SBTs, done during June 2000 to June 2006, have been analyzed retrospectively to assess the credibility of the method as a method of choice in selective high fertile group of patients. Women between 28 and 42 years have been included in the retrospective analysis, who had adequate number of eggs for fertilization, between 6 and 12.

**RESULTS AND CONCLUSIONS:**

Grade I blastocyst transfer resulted in 46.6% of clinical pregnancy and grade II blastocyst transfer resulted in 17.4% of clinical pregnancy rates. Overall pregnancy rate was 64%. Pregnancy loss, as early and late fetal wastages, was 11.06%.

Single embryo transfer, is becoming a well-accepted concept, which reduces the problems of multifetal gestation, though there are meager opportunities for monozygotic twinning. Multifetal gestation increases the obstetric risk into many folds. This includes premature delivery, small for gestational age and increased risk of congenital malformations.[1]

Many perspective randomized clinical studies support embryo culture up to 5 days till it reaches blastocyst stage to improve implantation rates. Single blastocyst transfer (SBT) is ideal in selected group of IVF cases, with at least five fertilized eggs and at least three grade I and grade II embryos on day 3.[1–5]

## MATERIALS AND METHODS

The aim of the study is to evaluate retrospectively, the outcome of SBT in selected group of patients, bringing out promising results for a period of 6 years - June 2000-June 2006 in our institution. Totally, 604 SBT cases have been analyzed.

Inclusion criteria for SBT:

1. Age between 28 and 42 years
2. Minimum of five fertilized eggs
3. Minimum - three, grade I and grade II embryos - on day 3
4. Couple who could able to understand the motive and scientific base of the extended embryo culture, was eligible for the day 5 ET.

Most of the couples agreed readily for the SBT. Only Grade IAA, Grade IAB, and Grade II AA were selected for transfer [Tables 1 and 2] while spare viable blastocysts were cryopreserved for future use. GnRH agonist (Suprefact)-long protocol was followed in 502 cases while rest of them had antagonist protocol using Orgalutron. The hormonal preparations used were: Recagon (Organon), Menopur (Ferring), Suprefact (Aventis), oragalutron (Organon), and Chorogon (Ferring). Most of them had only Recagon 200 IU per day till sixth day, followed by addition of Menopure 75 IU as per the requirement of the individual. Doses were subjected for small alterations depending on the nature of the cases, age, weight of the woman and the experience (ovarian response) of them in the previous attempt. Polycystic ovarian disease ovarian hyperstimulation syndrome cases and women with history of ovarian hyper stimulation syndrome (OHSS) had lesser doses with antagonist protocol.

**Table 1:** Gardner and Trounson's criteria of blastocyst scoring

Good G I	Fully expanded blastocyst/hatching blastocyst
Adequate G II	Fully expanded blastocyst, hatching D6 or moderate expansion D5
Mediocre G III	Moderate expansion D6/Early cavitation D5
Poor G IV	Early cavitation D6/Morula D5/D6

**Table 2:** ICM and TE scoring - Gardner and Trounson's criteria[7]

Good-A	High cell no; good cell to cell adhesion
Mediocre-B	Lower cell no; poor cell to cell attachment
Poor-C	No cell apparent - sparse ICM Granular, low cell no TE

Vitrolife, Sweden - sequential embryo culture media is used for all of them. The embryos were cultured in droplets under oil in trigas incubator (Nuair, USA) with 5% O_2_, 6% CO_2_ and 89% N_2_. Utensils and disposables were from Falcon, UK. Eppendorf microtips and pipett handles were also used for handling the embryos, in addition to the routine Pastuer pipettes from Falcon. Blastocysts with comparatively thicker zona and embryos of women more than 35 were subjected for Laser-assisted hatching using Saturn Laser system, UK, before ET.

Embryo transfer was cancelled if quality of blastocyst had not reached Grade I or Grade IIAA [Figures 1 -12 different stages and grades of embryo]. There were separate data maintained for cryopreserved embryos and transfer and not included in this analysis.

**Figure 1:**
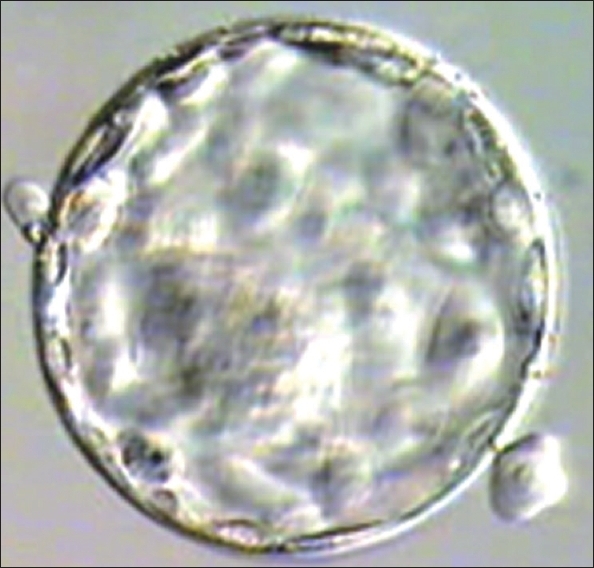
Blastocyst grade I AB

**Figure 2:**
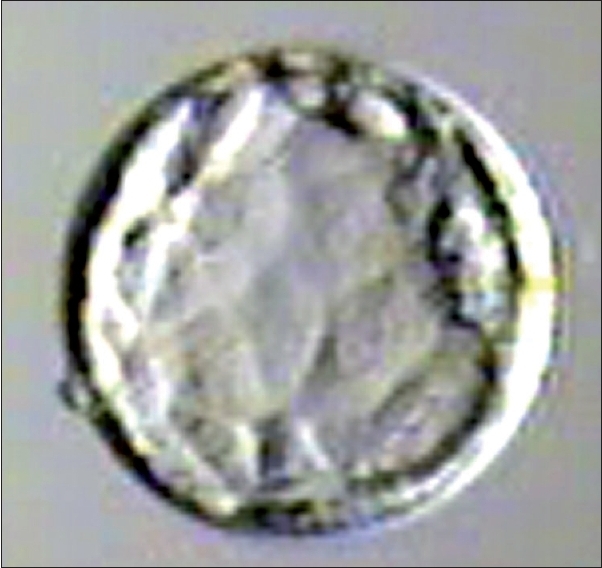
Blastocyst grade IIBA

**Figure 3:**
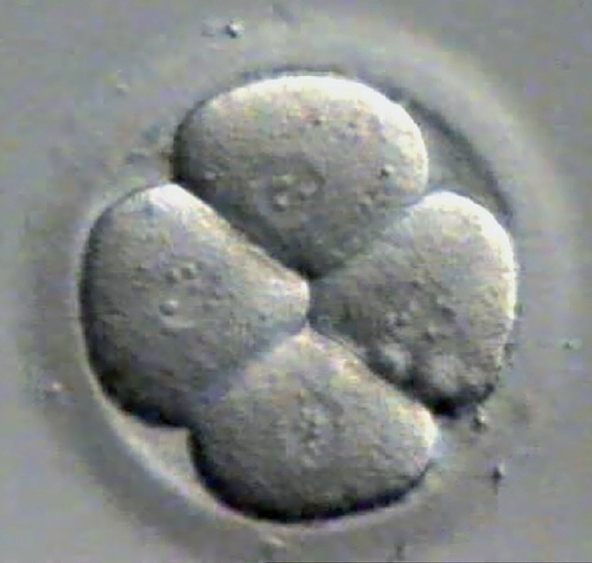
Day 2 grade I

**Figure 4:**
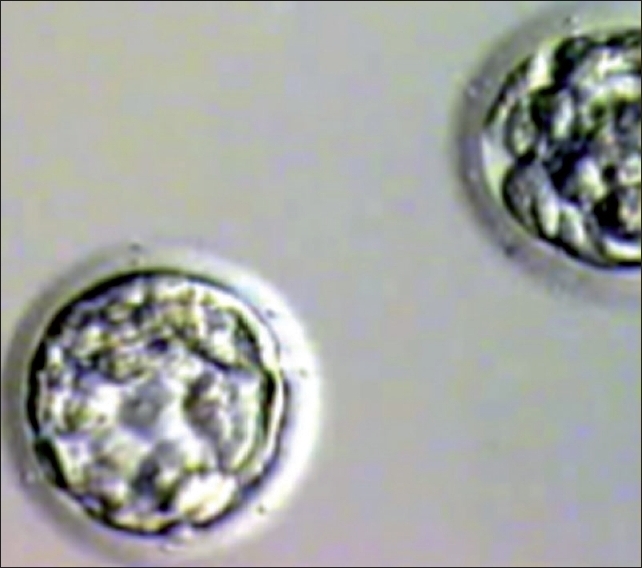
Blastocyst grade III

**Figure 5:**
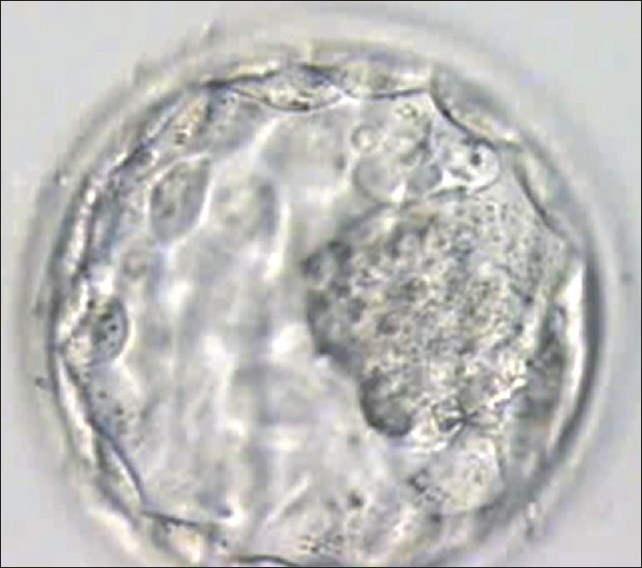
Expanding blastocyst grade I AA

**Figure 6:**
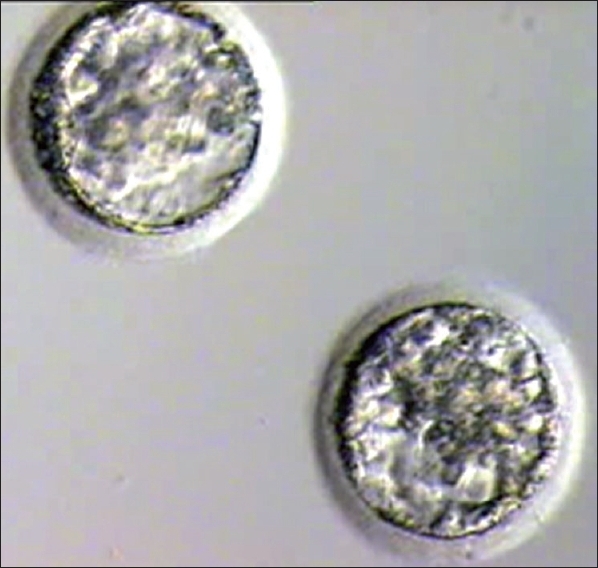
Blastocyst grade IV day 6

**Figure 7:**
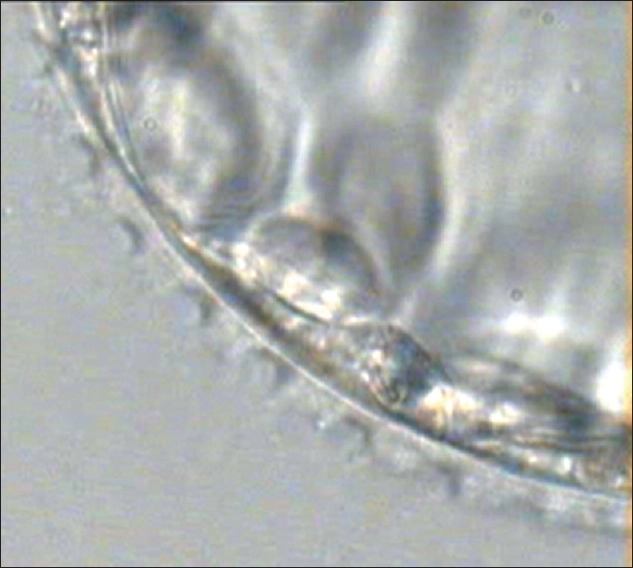
Blastocyst – laser assisted hatching

**Figure 8:**
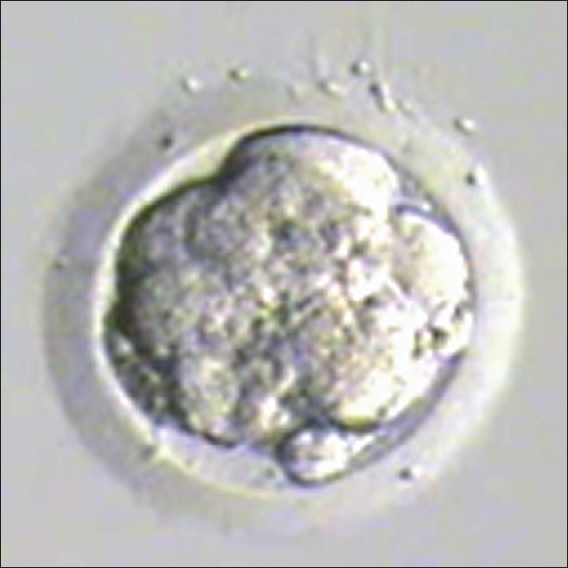
Morula

**Figure 9:**
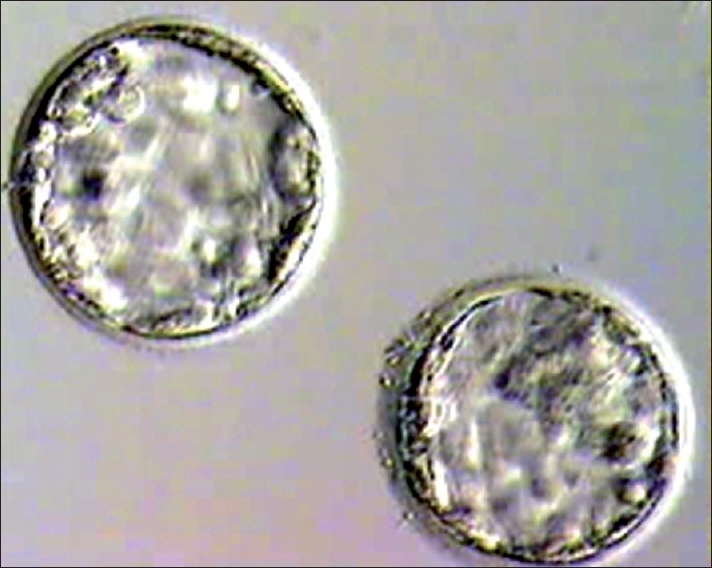
Blastocyst grade II A

**Figure 10:**
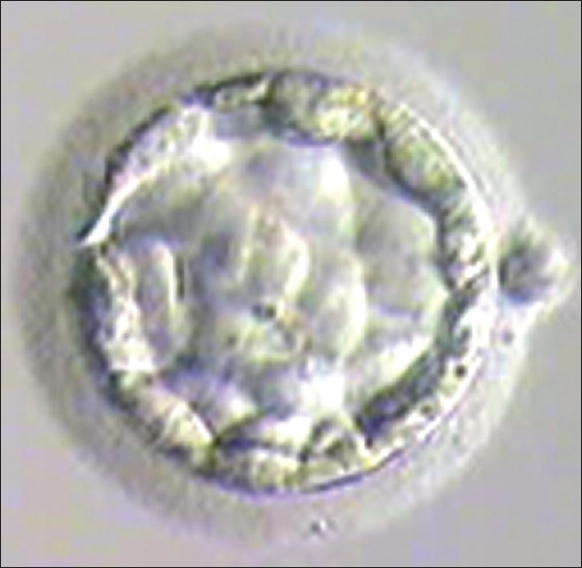
Early blastocyst

**Figure 11:**
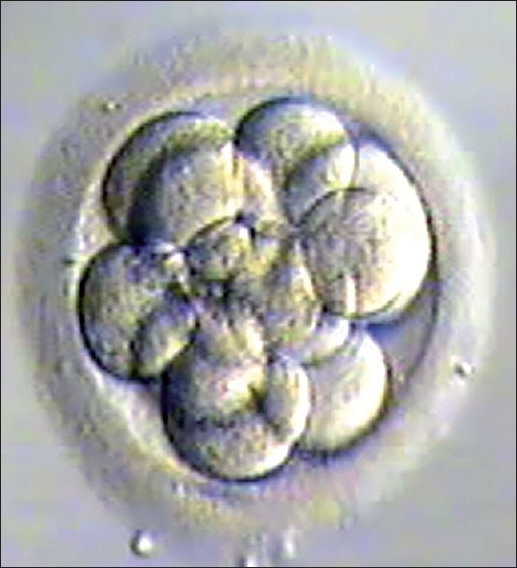
Day 3 grade I

**Figure 12:**
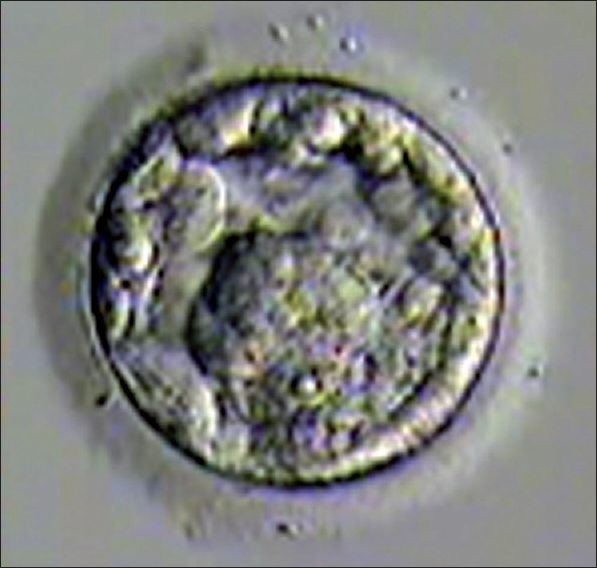
Blastocyst grade II AA

Blastocyst quality scoring was done using Gardner and Trounson's criteria [Tables 1 and 2]. Even early hatching blastocysts were taken as grade-I embryo. Cook-K-soft Embryo transfer catheter used for blastocyst transfer with or without ultrasound guidance as per the need. Luteal support was given with Inj. Progesterone (Gestone 50 mgm) in oil everyday. Inj. Pregnyl 1500 IU given on day 5, 9, 13, after ovum retrieval. Even mild cases of OHSS were detained from getting HCG supplementation. Serum Beta HCG levels assessed 15 days after ET. Positive Intrauterine gestational sac observed from 18th day itself in most of the cases. Totally six tubal ectopics reported, out of which four were amenable for methotrexate injections and two cases underwent laparoscopic correction.

## RESULTS

Mean age 32.9 years (SD 3.9 years, median 32.3) and 207 women were between 36 and 42 years. In most of the cases, it was II or III attempt IVF cycle. Laser-assisted hatching was done in 63.1% cases. Surplus blastocysts were cryopreserved in 28% of cases (either grades I or II). Serum Beta HCG level is analyzed, 15 days after ET. Positive test report, which gave clinical pregnancies, differs between 540 and 2150 IU depending on the sac size. Positive Intrauterine gestational sac observed from 18th day itself in most of the cases. Beta HCG levels below 540 mIU ended in either chemical pregnancies or blighted ovum without establishing the embryonic heartbeat. Totally six tubal ectopics reported, out of which four were amenable for methotrexate injections and two cases underwent laparoscopic correction. The causes of infertility for the study group of couples are, Tubal infertility, ovulatory dysfunctions (PCOD, Premature/Primary Ovarian failure and less ovarian reserve), Idiopathic Infertility, Uterine problems corrected (after fibroid/septum resection) and Surrogacy and Male infertility (ICSI done). Incidence of ICSI being 21.9% of the cases. Ovum donation done for 22.6% of cases with genuine ovum donors. Over all male infertility as an indication for this ART work being 38%.

**Chart 1:**
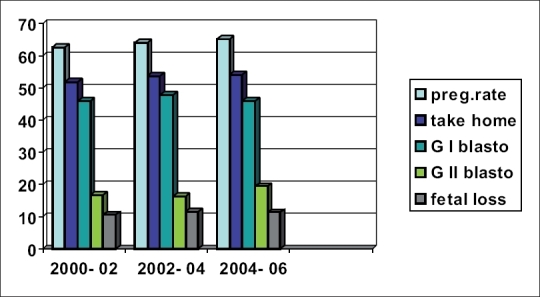
Outcome of Selective Single Embryo Transfer from year 2000-2006

Clinical pregnancy rate for this SBT analysis is 64.1 and 53.2% was the take home baby rate [Table 3 and chart 1]. Only 3% had monozygotic twinning which is acceptable, when compared with overall twinning rate if more than one embryo were transferred (33.3%). Early and late pregnancy loss was calculated as 11.06%. Clinical pregnancy rate is significantly higher with grade I transfers (46.6%) when compared with grade II blastocyst transfer (17.4%). When two blastocysts were transferred the clinical pregnancy rate was higher (70.1%) with more maternal and fetal morbidities and significant late fetal loss due to multiple gestations, ended with more or less similar take home baby rates as SBT. Clinical pregnancy rate of 36.6%, early and late fetal loss of 10.8% and take home baby rate of 25.8%, were observed in day 3 embryo transfer cases during this period.

**Table 3:** Data used for chart- Overall pregnancy rates, take home baby rates, Pregnancy rates when grade -I blastocysts transferred, Pregnancy rates when grade II blastocysts transferred and fetal loss highlihgted

Year	PR*	THBR%†	PR - G I blastocysts‡	PR - G II Blastocysts§	Fetal loss (%)
2000-2002	62.7	51.8	46.1	16.6	10.5
2002-2004	64.2	53.7	47.9	16.3	11.5
2004-2006	65.3	54.1	45.9	19.4	11.2

* Overall pregnancy rate

† Take home baby rate

‡ Pregnancy rate-when Grade I blastocyst transferred

§ Pregnancy rate when GII blastocyst transferred

## DISCUSSION

Most of the cases selected for the retrospective analysis were Idiopathic infertility with good ovarian reserve. The basic requirement for the extended embryo culture up to 5 days, being the good number of eggs (minimum 6) with good maturity and the above-mentioned inclusion criteria were fulfilled in the selected group of cases for retrospective analysis.

The twin rate, when two blastocysts were transferred were 33.3% as per the previous study reports, while this retrospective analysis has shown only 3% incidence of monozygotic twinning. The main outcome of this observational study was, significant increase in viable pregnancy rates and birth rates with SBT in a selected group of IVF patients, which is very much encouraging. It could be taken as an ideal embryo culture strategy in those mentioned group of highly fertile couple, to augment the success opportunities. Apart from the advantage of increased clinical pregnancy rates (64.1%), it reduces the multifetal gestation (3%) rates, thereby reducing the morbidities.

Earlier studies [6,7] also confirmed more or less similar benefits out of SBT. The ESHRE[8] also recommends SET in selected group of patients to reduce the incidence of twins perhaps fewer than 10%.

Certain laboratory conditions are mandatory for undertaking blastocyst cultures as the primary system of the laboratory. It necessitates expertise, optimal culture conditions and adequate experience in this sequential culture system. It also warrants skillful assessment of the blastocyst grading before transfer and correct timing of embryo transfer. Comparatively, blastocyst-microscopical quality evaluation is easier than D2 or D3 assessment and it comes in practice to choose the most viable blastocyst at once [Tables 1 and 2]. The probable reason for the augmented success opportunities may be:

1. We choose the most viable embryo at the end of day 5 (survival of the fittest) for transfer
2. Easier microscopical assessment possible with short exposure time
3. The technique offers better synchronization between embryo and endometrium to have healthy embryo maternal dialogue

There are more than 10 perspective randomized controlled trials comparing day 3 and day 5 transfers, supporting the blastocyst transfer on day 5. [9–15] When SBT programme is undertaken, the rest of the grade I or grade II blastocysts of the couple were cryopreserved. The most crucial event, being the ability to freeze such blastocysts with fairly good pregnancy rates in thaw cycles.

In our study, 89.8% of the couples produced at least one grade I or grade II blastocyst for SBT, most often with grade I blastocyst, while 10.2% of the couples had none for ET. But in a previous study,[16] only 80% of cases had successful blastocyst formation on day 5 of the culture. Encouraging higher figures were also reported.[17] This SBT helps in avoiding multifetal gestation, offering highly beneficial cost-effective management for infertility in selected group of couples who have adequate number of mature oocytes and in Ovum donation programmes.
